# Anti-helminthic niclosamide inhibits Ras-driven oncogenic transformation via activation of GSK-3

**DOI:** 10.18632/oncotarget.16255

**Published:** 2017-03-16

**Authors:** Sung Yong Ahn, Ji Hye Yang, Nam Hee Kim, Kyungro Lee, Yong Hoon Cha, Jun Seop Yun, Hee Eun Kang, Yoonmi Lee, Jiwon Choi, Hyun Sil Kim, Jong In Yook

**Affiliations:** ^1^ Department of Oral Pathology, Oral Cancer Research Institute, Yonsei University College of Dentistry, Seoul 03722, Korea; ^2^ Bioinformatics and Molecular Design Research Center, Yonsei University, Seoul 03722, Korea; ^3^ Department of Biotechnology, College of Life Science and Biotechnology, Yonsei University, Seoul 03722, Korea

**Keywords:** niclosamide, Ras oncogene, GSK-3, epithelial-mesenchymal transition (EMT)

## Abstract

Despite the importance of Ras oncogenes as a therapeutic target in human cancer, their ‘undruggable’ tertiary structures limit the effectiveness of anti-Ras drugs. Canonical Wnt signaling contributes to Ras activity by glycogen synthase kinase 3 (GSK-3)-dependent phosphorylation at the C-terminus and subsequent degradation. In the accompanying report, we show that the anti-helminthic niclosamide directly binds to GSK-3 and inhibits Axin functions in colon cancer cells, with reversion of Snail-mediated epithelial-mesenchymal transition. In this study, we report that niclosamide effectively suppresses Ras and nuclear NFAT activities regardless of the mutational status of Ras at nM levels. Mechanistically, niclosamide increased endogenous GSK-3 activity, shortening the half-life of mutant Ras. Further, niclosamide activates Raf-1 kinase inhibitory protein, a downstream target of Snail repressor. Niclosamide treatment attenuates Ras-induced oncogenic potential *in vitro* and *in vivo*. These findings provide a clinically available repositioned Ras inhibitor as well as a novel strategy for inhibiting the Ras via GSK-3.

## INTRODUCTION

Gain-of-function mutations (mostly located at the N-terminus) in Ras genes are commonly observed in human carcinogenesis as well as in therapeutic resistance [1, 2]. The oncogenic mutation of K-Ras is found in 25-30% of all cancer screened [3], and its emergence from wild type after targeted therapy provides acquired therapeutic resistance [4, 5]. While the mutant Ras are a promising therapeutic target in human cancer, there are no structurally defined surface pockets suitable for drug binding. Recently, mutation specific Ras-GTP-Raf binding has emerged for therapeutic targeting of Ras [6, 7], although a clinically available drug attenuating Ras activity is not yet available.

The oncogenic Ras mutations and genetic alterations in other MAPK/ERK signaling pathways, such as BRAF, are mutually exclusive events in human cancers, indicating that gain-of-Ras activity is essential for tumorigenesis [8]. Moreover, reciprocal regulations between the Ras activation and canonical Wnt pathways merit special attention. Indeed, K-Ras mutation stimulates canonical Wnt activity through inhibition of GSK-3 [9]. Similar to Snail and β-catenin in the canonical Wnt pathway [10], the Ras harbors a highly conserved destruction motif that is phosphorylated by GSK-3 and subsequently degraded [11]. Raf-1 kinase inhibitory protein (RKIP), an endogenous inhibitor of Ras signaling pathways, is directly suppressed by Snail, a key inducer of epithelial-mesenchymal transition (EMT) [12, 13]. Furthermore, transcriptional activity of nuclear factor of activated T cells (NFAT) is activated by the Ras/Raf pathway and is antagonized by GSK-3 activity [14]. Given the important role of GSK-3 in oncogenic signaling including in the Snail-mediated EMT program and canonical Wnt pathway [10, 15, 16], we hypothesized that modulation of GSK-3 activity by small molecules provides an alternative strategy for attenuating oncogenic Ras in human cancer.

Niclosamide is an oral salicylanilide derivative that has been FDA-approved since 1960 for use in the treatment of various tapeworm infections. Recently, niclosamide has emerged in anti-cancer therapeutics for various types of cancer [17, 18]. While many studies have demonstrated that niclosamide is involved in oncogenic signaling pathways such as Wnt, ROS, Notch, mTOR and autophage [17–19], its molecular target and mode of action is not well-understood. In the accompanying report, we show that niclosamide directly targets the Axin-GSK-3 binding site, resulting in suppression of canonical Wnt activity and the Snail-mediated EMT program of colon cancer cells. We report that niclosamide increased cellular GSK-3 activity and accelerated Ras degradation regardless of mutational status. These results provide a clinically available drug and a novel strategy for regulating oncogenic Ras.

## RESULTS AND DISCUSSION

### Niclosamide suppresses Ras abundance in a GSK-3-dependent manner

**Figure 1:**
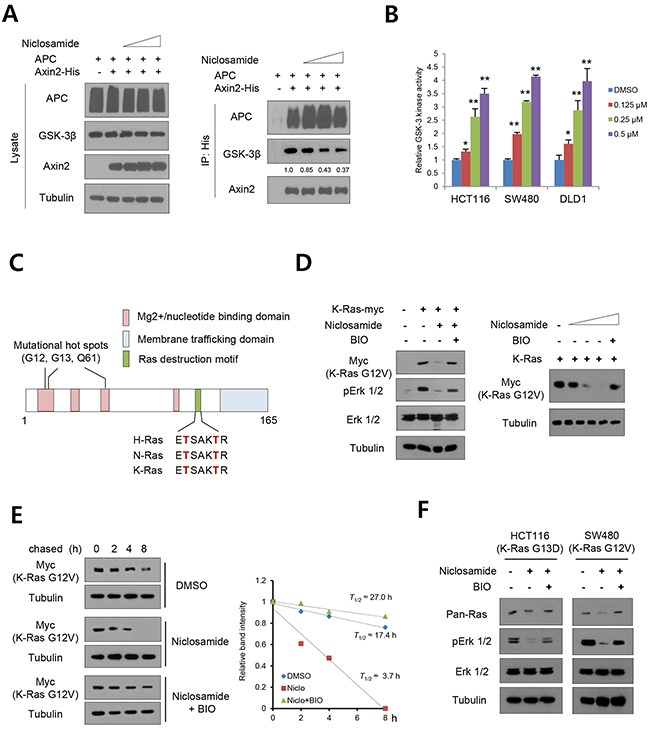
GSK-3-dependent Ras suppression by niclosamide **(A)** The 293 cells were transiently transfected with His-tagged Axin2 and wild type APC expression vectors, then treated with increasing doses of niclosamide for an 8h period followed by immunoblot analysis of whole cell lysates (lysate). The GSK3 binding activities in lysate were determined by Ni-NTA bead immunoprecipitation (IP) followed by immunoblot analysis for endogenous GSK3 and APC. Relative binding of GSK-3 to Axin2 input was determined by ImageJ program. **(B)** The dose-dependent endogenous GSK-3 kinase activity in colon cancer cells was measured. The relative kinase activity was determined from triplicated experiments. Data are expressed as means and s.d. The double asterisks denote *p* < 0.01 by Student's t-tests. **(C)** Schematic diagram of structural domains of Ras. Conserved phosphorylation residues by GSK-3 are shown in red. **(D)** The 293 cells transfected with empty vector or myc-tagged K-Ras G12V mutant expression vector were treated with niclosamide (0.25 μM) or in combination with BIO (1 μM) for 18 h. Protein abundance of K-Ras (myc), total Erk 1/2 (Erk), and phospho-Erk 1/2 (pErk) were determined by immunoblotting (left panels). The mutant K-Ras abundance was determined with increasing dose of niclosamide (0, 0.125, 0.25, 05 μM) in combination with BIO (right panels). **(E)** Mutant K-Ras half-life in control or niclosamide or in combination with BIO was determined by pulse-chase analysis and immunoblotting (left panels). The half-life of mutant K-Ras was determined from the slope of densitometric protein abundance (right panel). **(F)** Colon cancer cells having mutant K-Ras were treated with chemicals as indicated for 18 h, and abundance of Ras, total Erk 1/2 (Erk), and phospho-Erk 1/2 (pErk) were determined by immunoblotting.

The GSK-3 is highly expressed in normal cells and is one of the few kinases inactivated following stimulation of exogenous growth factors and oncogenic signaling [20]. For example, canonical Wnt suppresses GSK-3 activity through the sequestration of soluble GSK-3 into multivesicular endosomes and knockdown of Axin2 increased nuclear GSK3 activity [21, 22]. Conversely, cellular GSK-3 inactivation in the mammary epithelium resulted in precancerous condition and development of adenocarcinoma [23]. In the accompanying report, we show that niclosamide directly targets the Axin-GSK-3 binding site. To re-validate Axin2-GSK-3-APC interaction, we overexpressed His-tagged Axin2 and wild type APC in 293 cells and treated niclosamide followed by immunoprecipitation analysis. Niclosamide treatment decreased GSK-3 interaction, but not APC binding, to Axin2 (Figure 1A). To directly examine whether niclosamide affects endogenous GSK-3 activity, the colon cancer cells were subjected to GSK-3 kinase assay following niclosamide treatment. Indeed, nM level of niclosamide was sufficient to increase endogenous GSK-3 activity in colon cancer cells in a dose-dependent manner (Figure 1B), indicating that disruption of Axin-GSK3 binding increased cellular GSK-3 kinase activity. Similarly to β-catenin and Snail, the Ras protein harbors a highly conserved destruction motif that is phosphorylated by GSK-3, after which the phosphorylated Ras is degraded by β-TrCP-directed ubiquitination and proteasomal activity [11]. Importantly, the destruction motif of Ras is located on the C-terminus while the mutational hot spot in human cancer is on the N-terminus [11, 24], providing an alternative approach to suppressing mutant Ras by modulation of GSK-3 (Figure 1C). To prove this hypothesis, we next chose the K-Ras-G12V mutant to test the possibility that increased GSK-3 activity by niclosamide suppresses mutant Ras. We then overexpressed myc-tagged Ras mutant in 293 cells and treated niclosamide in combination with GSK-3 specific inhibitor BIO. Indeed, protein abundance of mutant K-Ras and endogenous phospho-ERK (extracellular signal-regulated kinase) were suppressed by niclosamide, while the GSK-3 inhibitor BIO attenuated the effects of niclosamide (Figure 1D). To further examine whether niclosamide decreases Ras stability, we transfected mutant K-Ras expression vector and chased Ras protein abundance under cycloheximide treatment. We found that the mutant K-Ras protein half-life was significantly shortened by niclosamide treatment and the pharmacological effect was largely recovered by potent GSK-3 kinase inhibitor BIO (Figure 1E). We next chose colon cancer cells having K-Ras mutation and treated niclosamide in combination with BIO. Indeed, 0.125 μM niclosamide was sufficient to suppress the mutant Ras-Erk pathway while BIO re-activated the Ras activity in colon cancer cells (Figure 1F). These results support that niclosamide decreases Ras stability via increasing GSK-3 activity in colon cancer cells.

### Niclosamide suppressed RAS activity in colon cancer cells regardless of mutational status

**Figure 2:**
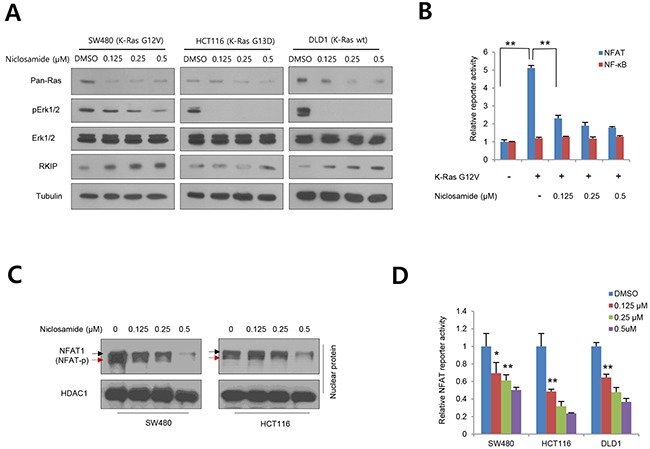
Niclosamide suppresses Ras activity at various levels **(A)** Colon cancer cells having mutant or wild type K-Ras treated with nM niclosamide for 18 h, and abundance of Ras, total Erk 1/2 (Erk), phospho-Erk 1/2 (pErk), and PKIP were determined by immunoblotting. Mutational status of K-Ras is noted on cell lines. **(B)** NFAT reporter activities in 293 cells were determined in K-Ras G12V transfected with a control (-) or increasing concentration of niclosamide. Non-related NF-ĸB reporter serves as negative control. **(C)** Nuclear abundance and phosphorylation of NFAT1 in colon cancer cells. HCT116 and SW480 cells were treated with increasing doses of niclosamide and NFAT1 abundance was measured with immunoblot analysis from nuclear fraction. Hyperphosphorylated and active dephosphorylated isoforms denote black and red arrows, respectively. HDAC1 serves as loading control for nuclear fraction. **(D)** NFAT reporter activities in colon cancer cells were determined with control or various concentration of niclosamide. The reporter activity in (B) and (D) was normalized to the co-transfected pRL-SV40-*renilla* activity from triplicate experiments. Data are expressed as means and s.d. The double asterisks denote *p* < 0.01; one asterisk denotes *p* < 0.05.

Although GSK-3 is a well-known key kinase in the canonical Wnt pathway [20, 25], the kinase activity is also closely involved in many aspects of the Ras pathway. For example, Snail protein stability and abundance are tightly controlled by GSK-3-mediated serial phosphorylation and subsequent proteasomal degradation [10, 15]. Intriguingly, Snail directly suppresses Raf-1 kinase inhibitory protein (RKIP), resulting in activation of the Raf-1/Mek/Erk pathway and EMT progression [13, 26, 27]. In addition, nuclear import and transcriptional activity of the nuclear factor of activated T-cells (NFAT) are activated by Ras/Raf pathway whereas they are suppressed by GSK-3 [28–30]. These observations suggest that GSK-3 involvement in many layers of Ras/Raf/Erk signaling. Extending our observations to the Ras/Raf/Erk pathway and NFAT transcriptional activity, we treated niclosamide on colon cancer cells having variable K-Ras status. As shown above, Ras protein abundance and ERK phosphorylation were significantly decreased by niclosamide treatment regardless of Ras mutations (Figure 2A). As a transcriptional target of Snail repressor, niclosamide increased RKIP abundance in a dose-dependent manner. To further examine whether niclosamide suppresses K-Ras-dependent NFAT transcriptional activity, we next co-transfected NFAT reporter having IL-2 promoter and mutant K-Ras in 293 cells in combination with niclosamide. Indeed, the reporter activity was increased by overexpression of mutant K-Ras, but was largely diminished by niclosamide, while non-related NF-ĸB reporter was minimally changed (Figure 2B). NFAT is phosphorylated by GSK-3 and translocated into the cytosolic space [28]. In colon cancer cells, niclosamide effectively decreased nuclear abundance along with increased phosphorylation (Figure 2C) and transcriptional activity of NFAT1 (also known as NFATp) in a dose-dependent manner (Figure 2D). These results support that niclosamide efficiently suppresses the Ras/Raf/Erk pathway and the Ras-responsible transcriptional program.

### Niclosamide inhibits Ras-induced transformation

**Figure 3:**
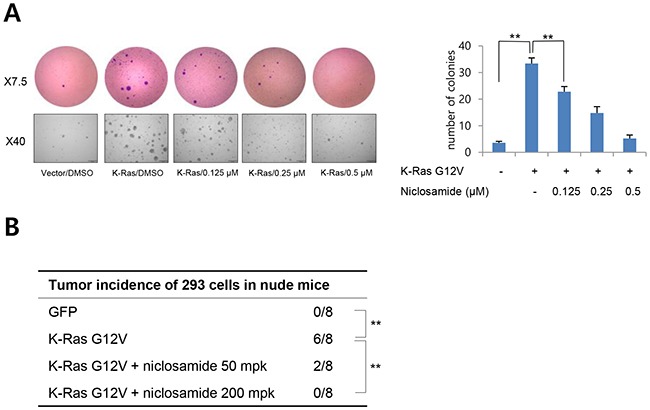
Niclosamide inhibits Ras-induced cell transformation **(A)** Representative colonies of soft agar assays as described in Methods (left panels). The data represent the colony formation ability of K-Ras transfected 293 cells treated with different concentrations of niclosamide. The colony number was counted from 5 separate fields (right panel). Results are shown as means and s.d. Two asterisks, P < 0.01. **(B)** Niclosamide inhibits the K-Ras-induced tumorigenic potential of 293 cells *in vivo*. The 293 cells were transiently transfected with vector or mutant K-Ras, and a suboptimal number of cells (5 × 10^5^) cells were inoculated into the flank of athymic nude mice prior to 24 h of niclosamide treatment. Vehicle or niclosamide in vehicle (50 mg/kg, 200 mg/kg) was intraperioneally administered. Two asterisks, P < 0.01 by Fisher's exact test.

To examine the functional relevance of niclosamide on Ras suppression, we transiently transfected mutant K-Ras and assayed anchorage-independent growth. Overexpression of mutant K-Ras gave rise to significantly higher numbers of larger colonies in soft agar than control vector transfectants, and niclosamide treatment suppressed anchorage-independent growth in a dose-dependent manner (Figure 3A). In the accompanying report, we show that niclosamide significantly suppressed the *in vivo* tumorigenic potential of colon cancer cells having mutations of APC (SW480) or β-catenin (HCT116) with K-Ras. To examine the functional roles of niclosamide on Ras oncogene, we next transiently transfected mutant K-Ras into 293 cells and assessed *in vivo* tumorigenic potential with niclosamide. Indeed, tumor initiating potential induced by mutant K-Ras was attenuated by administration of niclosamide (Figure 3B). These results support that niclosamide efficiently suppresses Ras activity *in vitro* and *in vivo*.

**Figure 4:**
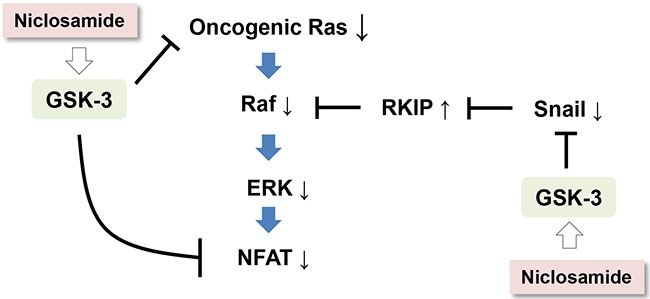
A schematic diagram depicting a potential mechanism by which niclosamide suppresses Ras activity in cancer cells

Together with activation of the canonical Wnt pathway, somatic mutations of small GTPase Ras isoforms are frequently found in human cancer, these genetic alterations being associated with resistance against standard or targeted therapeutics [4, 5]. Despite its importance as an anticancer target, Ras protein is hardly targetable by small molecules owing to the absence of druggable pockets on its surface. In this study, we provide evidence that increased GSK-3 activity by repositioned niclosamide efficiently suppresses Ras activity regardless of its mutational status via increased GSK-3 activity in colon cancer cells (Figure 4). Our observations suggest an alternative strategy as well as a clinically available drug for regulation of Ras in human cancer.

## MATERIALS AND METHODS

### Cell lines and reagents

Colon cancer cell lines (HCT116, SW480 and DLD1) and 293 cells were from ATCC and were maintained under conditions recommended by the provider. All cell lines were verified using DNA fingerprinting analysis with short tandem repeat markers. The niclosamide was purchased from Cayman, GSK-3 specific inhibitor BIO (6-bromoindirubin-3′-oxime, B1686) was purchased for Sigma, and chemicals were solubilized in DMSO for *in vitro* experiments. K-Ras G12V mutant was kindly provided by Dr. K. H. Chun and its sequence was verified for experiments. NFAT reporter having IL-2 promoter was obtained from Addgene (plasmid #10959) and NF-ĸB reporter as a negative control was purchased from Agilent Technologies (219078). Expression vector for wild type APC was obtained from Addgene (#16507).

### GSK-3 kinase assay, reporter assay, immunoblot assay, immunoprecipitation, and pulse chase experiment

GSK-3 kinase activity in whole cells was performed using the GSK-3 kinase assay kit (ENZO Biochem) as described previously [16]. For reporter assay, cells were transfected with 100 ng of reporter gene constructs with 1 ng of pRL-SV40-*Renilla* vector. Reporter activities were measured with a dual luciferase assay system (Promega) 48 h after transfection and normalized by measuring co-transfected *renilla* activity. Reporter gene activities are presented as relative light units to those obtained from negative DMSO control. For the western blot analyses, protein extracts were prepared in Triton X-100 lysis buffer. Antibodies against pan Ras (sc16691, Santa Cruz, 1:2,000), Erk1/2 (4695S, Cell Signaling, 1:1,000), pErk1/2 (4377S, Cell Signaling, 1:1,000), RKIP (sc376925, Santa Cruz, 1:1,000), NFAT-p (sc7296, Santa Cruz, 1:500), myc (LF-MA0046, Ab Frontier, 1: 5,000) and Tubulin (LF-PA0146A, Ab Frontier, 1:5,000) were obtained from the commercial vendors. For immunoprecipitation assay, His-tagged Axin2 expression vector and wild type APC were transiently transfected into 293 cells. After a 48 h period, the cells were treated with different dose of niclosamide for 8 h, and whole cell Triton X-100 lysates were incubated with Ni-NTA beads (Invitrogen). The recovered proteins were resolved by SDS-PAGE and subjected to immunoblot analysis for GSK3, APC, and input (1/20 volume) control. The pulse-chase analyses of K-Ras mutant were performed as described previously [15]. In brief, 293 cells were transfected with myc-tagged K-Ras G12V expression vector for 48 h. The transfected cells were treated with cycloheximide (50 μg/ml), followed by chase for the indicated time periods in the presence or absence of niclosamide (0.25 μM) and BIO (1 μM). Cell lysates were harvested and subjected to immunoblot analysis against myc-tag. The band intensity was measured with densitometer, and relative intensity compared to loading control was calculated with ImageJ program provided by NIH. The nuclear abundance of NFAT was determined from nuclear-cytosolic fractionation with hypotonic buffer solution. Briefly, the colon cancer cells (1 × 10^6^) were collected into microcentrifuge tubes. The PBS-washed cells were treated with 400 μl of hypotonic buffer (10 mM HEPES, pH7.9; 10 mM KCl; 1 mM DTT with protease inhibitors) on ice for 5 min. The cell membrane was ruptured by adding 10% NP-40 to a final concentration of 0.6%, then vigorously vortexed for 10 sec followed by high-speed centrifuge for 30 sec. The supernatant cytosolic fractions were collected separately, and nuclear pellets were washed with ice-cold PBS twice. Nuclear protein was extracted with hypertonic buffer (20 mM HEPES, pH7.9; 0.4 M NaCl; 1mM DTT with protease inhibitors) for 15 min on ice followed by high-speed centrifuge.

### Soft agar assay

293 cells transiently transfected with empty vector or K-Ras G12V mutant were suspended at 1 × 10^4^ cells per 6-well plate with 1 ml of 0.3% low-melting agar in 2X DMEM containing 20% FBS and overlaid on a layer of 1 ml of 1% agar in the same medium. Two weeks later, colonies were visualized by staining with 0.05% crystal violet in 10% ethanol for 30 min and viable colonies that contained > 50 cells were counted from 5 fields with a stereomicroscope. Representative colonies were photographed and 2 independent experiments were performed.

### Animal experiment

All animal experiments were performed in accordance with the Institutional Animal Care and Use Committee of Yonsei University and approved by the Animal Care Committee of the Yonsei University College of Dentistry. Female athymic nude mice (6 weeks old) were used for xenograft assays. The 293 cells were transiently transfected with vector control or mutant K-Ras G12V prior to 48 h of *in vivo* inoculation. A suboptimal number of the cells (5 × 10^5^) were resuspended in 100 μl of PBS and injected into flank subcutaneous tissue. For intraperitoneally administration, vehicle (10% Cremophor EL and 0.9% NaCl) or niclosamide in vehicle (50 mg/kg, 200 mg/kg) were injected daily (6 days/week), and the mice were monitored daily and weighed twice weekly for 6 weeks.

### Statistics

All statistical analysis of *in vitro* study was performed with two-tailed Student's t-tests; data are expressed as means and s.d. The double asterisks denote P < 0.01; one asterisk denotes P < 0.05. No statistical method was used to predetermine sample size.

## References

[1] Parada LF, Tabin CJ, Shih C, Weinberg RA (1982). Human EJ bladder carcinoma oncogene is homologue of Harvey sarcoma virus ras gene. Nature.

[2] Downward J (2003). Targeting RAS signalling pathways in cancer therapy. Nat Rev Cancer.

[3] Karnoub AE, Weinberg RA (2008). Ras oncogenes: split personalities. Nat Rev Mol Cell Biol.

[4] Karapetis CS, Khambata-Ford S, Jonker DJ, O–Callaghan CJ, Tu D, Tebbutt NC, Simes RJ, Chalchal H, Shapiro JD, Robitaille S, Price TJ, Shepherd L, Au HJ (2008). K-ras mutations and benefit from cetuximab in advanced colorectal cancer. N Engl J Med.

[5] Diaz LA, Williams RT, Wu J, Kinde I, Hecht JR, Berlin J, Allen B, Bozic I, Reiter JG, Nowak MA, Kinzler KW, Oliner KS, Vogelstein B (2012). The molecular evolution of acquired resistance to targeted EGFR blockade in colorectal cancers. Nature.

[6] Ostrem JM, Peters U, Sos ML, Wells JA, Shokat KM (2013). K-Ras(G12C) inhibitors allosterically control GTP affinity and effector interactions. Nature.

[7] Shima F, Yoshikawa Y, Ye M, Araki M, Matsumoto S, Liao J, Hu L, Sugimoto T, Ijiri Y, Takeda A, Nishiyama Y, Sato C, Muraoka S (2013). In silico discovery of small-molecule Ras inhibitors that display antitumor activity by blocking the Ras-effector interaction. Proc Natl Acad Sci U S A.

[8] Fernandez-Medarde A, Santos E (2011). Ras in cancer and developmental diseases. Genes Cancer.

[9] Li J, Mizukami Y, Zhang X, Jo WS, Chung DC (2005). Oncogenic K-ras stimulates Wnt signaling in colon cancer through inhibition of GSK-3beta. Gastroenterology.

[10] Yook JI, Li XY, Ota I, Fearon ER, Weiss SJ (2005). Wnt-dependent regulation of the E-cadherin repressor snail. J Biol Chem.

[11] Jeong WJ, Yoon J, Park JC, Lee SH, Lee SH, Kaduwal S, Kim H, Yoon JB, Choi KY (2012). Ras stabilization through aberrant activation of Wnt/beta-catenin signaling promotes intestinal tumorigenesis. Sci Signal.

[12] Shin SY, Rath O, Choo SM, Fee F, McFerran B, Kolch W, Cho KH (2009). Positive- and negative-feedback regulations coordinate the dynamic behavior of the Ras-Raf-MEK-ERK signal transduction pathway. J Cell Sci.

[13] Beach S, Tang H, Park S, Dhillon AS, Keller ET, Kolch W, Yeung KC (2008). Snail is a repressor of RKIP transcription in metastatic prostate cancer cells. Oncogene.

[14] Flockhart RJ, Armstrong JL, Reynolds NJ, Lovat PE (2009). NFAT signalling is a novel target of oncogenic BRAF in metastatic melanoma. Br J Cancer.

[15] Yook JI, Li XY, Ota I, Hu C, Kim HS, Kim NH, Cha SY, Ryu JK, Choi YJ, Kim J, Fearon ER, Weiss SJ (2006). A Wnt-Axin2-GSK3beta cascade regulates Snail1 activity in breast cancer cells. Nat Cell Biol.

[16] Lee DG, Kim HS, Lee YS, Kim S, Cha SY, Ota I, Kim NH, Cha YH, Yang DH, Lee Y, Park GJ, Yook JI, Lee YC (2014). Helicobacter pylori CagA promotes Snail-mediated epithelial-mesenchymal transition by reducing GSK-3 activity. Nat Commun.

[17] Osada T, Chen M, Yang XY, Spasojevic I, Vandeusen JB, Hsu D, Clary BM, Clay TM, Chen W, Morse MA, Lyerly HK (2011). Antihelminth compound niclosamide downregulates Wnt signaling and elicits antitumor responses in tumors with activating APC mutations. Cancer Res.

[18] Sack U, Walther W, Scudiero D, Selby M, Kobelt D, Lemm M, Fichtner I, Schlag PM, Shoemaker RH, Stein U (2011). Novel effect of antihelminthic Niclosamide on S100A4-mediated metastatic progression in colon cancer. J Natl Cancer Inst.

[19] Wieland A, Trageser D, Gogolok S, Reinartz R, Hofer H, Keller M, Leinhaas A, Schelle R, Normann S, Klaas L, Waha A, Koch P, Fimmers R (2013). Anticancer effects of niclosamide in human glioblastoma. Clin Cancer Res.

[20] Kaidanovich-Beilin O, Woodgett JR (2011). GSK-3: Functional Insights from Cell Biology and Animal Models. Front Mol Neurosci.

[21] Taelman VF, Dobrowolski R, Plouhinec JL, Fuentealba LC, Vorwald PP, Gumper I, Sabatini DD, De Robertis EM (2010). Wnt signaling requires sequestration of glycogen synthase kinase 3 inside multivesicular endosomes. Cell.

[22] Wu ZQ, Brabletz T, Fearon E, Willis AL, Hu CY, Li XY, Weiss SJ (2012). Canonical Wnt suppressor, Axin2, promotes colon carcinoma oncogenic activity. Proc Natl Acad Sci U S A.

[23] Dembowy J, Adissu HA, Liu JC, Zacksenhaus E, Woodgett JR (2015). Effect of glycogen synthase kinase-3 inactivation on mouse mammary gland development and oncogenesis. Oncogene.

[24] Prior IA, Lewis PD, Mattos C (2012). A comprehensive survey of Ras mutations in cancer. Cancer Res.

[25] Cohen P, Frame S (2001). The renaissance of GSK3. Nat Rev Mol Cell Biol.

[26] Yeung K, Seitz T, Li S, Janosch P, McFerran B, Kaiser C, Fee F, Katsanakis KD, Rose DW, Mischak H, Sedivy JM, Kolch W (1999). Suppression of Raf-1 kinase activity and MAP kinase signalling by RKIP. Nature.

[27] Shin SY, Rath O, Zebisch A, Choo SM, Kolch W, Cho KH (2010). Functional roles of multiple feedback loops in extracellular signal-regulated kinase and Wnt signaling pathways that regulate epithelial-mesenchymal transition. Cancer Res.

[28] Beals CR, Sheridan CM, Turck CW, Gardner P, Crabtree GR (1997). Nuclear export of NF-ATc enhanced by glycogen synthase kinase-3. Science.

[29] Ichida M, Finkel T (2001). Ras regulates NFAT3 activity in cardiac myocytes. J Biol Chem.

[30] Al-Mulla F, Bitar MS, Al-Maghrebi M, Behbehani AI, Al-Ali W, Rath O, Doyle B, Tan KY, Pitt A, Kolch W (2011). Raf kinase inhibitor protein RKIP enhances signaling by glycogen synthase kinase-3beta. Cancer Res.

